# Preclinical Evaluation of Ureidosulfamate Carbonic Anhydrase IX/XII Inhibitors in the Treatment of Cancers

**DOI:** 10.3390/ijms20236080

**Published:** 2019-12-02

**Authors:** Kaye J. Williams, Roben G. Gieling

**Affiliations:** 1 Faculty of Biology, Medicine and Health, School of Pharmacy and Optometry, University of Manchester, Manchester M13 9PL, UK; kaye.williams@manchester.ac.uk; 2 Faculty of Health and Life Sciences, Department of Applied Sciences, University of Northumbria, Newcastle Upon Tyne NE1 8ST, UK

**Keywords:** carbonic anhydrases (CAs), ureido-substituted benzenesulfonamide, ureido-substituted sulfamate, CAIX/XII inhibitors, hypoxia, S4, U-104, SLC-0111

## Abstract

Carbonic anhydrases (CAs) are a family of enzymes involved in the pH regulation of metabolically active cells/tissues. Upregulation of the CAIX/XII isoforms is associated with hypoxic tumours and clinically linked with malignant progression, treatment resistance and poor prognosis. The elucidation of the crystal structure of the catalytic domains of CAIX/XII provided the basis for the generation of CAIX/XII selective inhibitors based on the sulfonamide, sulfamate and coumarins chemical structures. Ureido-substituted benzenesulfonamide CAIX/XII inhibitors have shown significant potential, with U-104 (SLC-0111) currently present in clinical Phase I/II. Ureido-substituted sulfamate CAIX/XII inhibitors have received less attention despite encouraging preclinical test results. In triple-negative breast cancer (TNBC), ureidosulfamates revealed a significant antitumour (FC9-398A) and antimetastatic potential (S4). In small cell lung cancer (SCLC), a cancer cell type very sensitive to a dysregulation in CAIX signaling, S4 treatment was particularly effective when combined with cisplatin with no evidence of acquired cisplatin-resistance. These successful anticancer strategies should provide a solid basis for future studies on ureido-substituted sulfamates.

## 1. Introduction to Carbonic Anhydrase IX/XII Inhibitors

Carbonic anhydrases (CA, EC 4.2.1.1) catalyse the reversible hydration of carbon dioxide to bicarbonate and protons [H_2_O + CO_2_ ↔ HCO_3_^−^ + H^+^]. The human alpha carbonic anhydrase family contains fifteen isoforms, three of which lack CA activity, of which the majority is expressed in all tissues/cells. The expression of CA isoform IX in normal tissues is restricted to the basolateral membrane of epithelial cells of the gastric, intestinal and gallbladder mucosa [1]. The expression of CA XII in normal tissues is much more widespread. In solid cancers, the upregulation of CA isoforms IX and XII is closely associated with hypoxia—the oxygen deprivation of cancer cells due to the combination of a poor tumour vascularisation and a high proliferation rate [2,3]. The overexpression of transmembrane proteins CAIX and CAXII in cancer tissues is associated with multiple markers of cancer progression including high tumour growth, tumour cell migration, infiltration of surrounding normal tissues and the formation of metastases [2,3,4]. There is a well-established role of CAIX in facilitating migration and invasion of cancer cells. Conditional shRNA-mediated silencing of *CA9* gene in HT-1080 (fibrosarcoma) cells, reduced the expression of proteins such as protein kinase 1 (ROCK1) involved in focal adhesions (FAs). Silencing of *CA 9* gene affected the assembly of these highly dynamic and multicomplex protein structures attached to the plasma membrane, resulting in a significant reduced spreading, migration and invasion of Matrigel [5]. In lamellipodia, membranous protrusions at the leading edge of migrating cells that provide direction to the cell movement [6], CAIX colocalised with paxillin in FAs [7]. CAIX is also an important component of invadopodia, F-actin-rich protrusions of the plasma membrane involved in extracellular matrix (ECM) proteolysis and invasion [6]. Swayampakula and colleagues demonstrated, using a proximity-dependent labelling approach in proteomics study (BiolD), that CAIX associates with β1 integrins and matrix metalloproteinase 14 (MMP14), both components of invadopodia. CAIX stimulated the activation of MMP14 by supplying protons required for MMP14 catalytic activity, enhancing the degradation of type I collagen and stimulating MDA-MB-231 (breast carcinoma) invasion [8]. CAIX expression is controlled by hypoxia/Hypoxia Inducible Factor 1 (HIF-1) via the binding of this transcription factor to the Hypoxia-Response Elements (HREs) in the 5’-upstream genomic region of *CA9* gene [3]. HIF-1 is a heterodimer that consists of a constitutively-expressed beta subunit (HIF-1β) and a hypoxia-induced alpha subunit (HIF-1α). In hypoxic conditions, HIF-1α is stabilised, allowing for HIF-1 activity to increase and stimulate the expression of various genes including CAIX. CAXII expression is also associated with the hypoxic environment in solid tumours despite the fact that HREs are essentially lacking in the 5’-upstream genomic region of *CA12* gene [2]. The role of CAXII in the interaction between the tumour cell and ECM is less well known and has not been linked with FAs. However, selective silencing of *CA12* gene in MDA-MB-231 cells (MDA-MB-231-siCAXII), interfered with the p38 mitogen-activated protein kinases (p38 MAPK) signalling pathway, which resulted in decreased cell migration and Matrigel-invasion [9]. Orthotopic tumours derived from MDA-MB-231-siCAXII cells were less metastatic than control cells. Silencing of the Hedgehog signaling pathway in MDA-MB-231 cells reduced CAXII expression and migration, which highlighted a potential role of CAXII in cancer cell migration [10].

**Figure 1:**
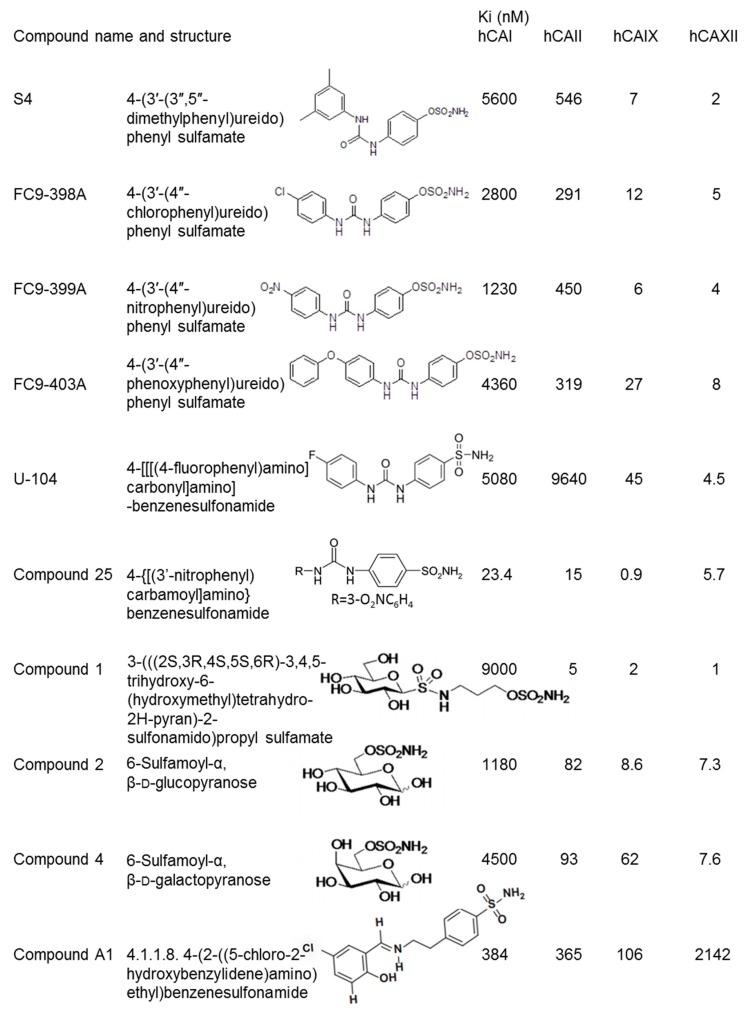
Chemical structures and Ki values for CAI, CAII, CAIX and CAXII for ureidosulfamates S4, FC9-398A, FC9-399A and FC9-403A, ureidobenzenesulfonamides U-104 (SLC-0111) and compound 25, carbohydrate-based sulfamate compounds 1, 2 and 4 and sulfonamide A1.

A decade ago, the elucidation of the crystal structure of the catalytic domain of CAIX provided the basis for the subsequent design of isoform-specific small molecular CA inhibitors based on sulfonamide, sulfamate and coumarin chemical structures [11]. These compounds have a high affinity for the tumour-associated CA-isoforms (CAIX/XII) over the cytosolic off-target isoforms (CAI/II). In particular, the ureido-substituted CAIX/XII inhibitors of the benzenesulfonamide and sulfamate chemical classes have proven effective in biological studies by different research groups. The flexibility that the ureido linker provides in these compounds is the determining factor in controlling the inhibitory power, allowing for the different R moieties to orientate in different subpockets of the active sites of the CA enzyme [12]. Ureido CAIX/XII inhibitors have both been intensively validated in 2D (monolayer) and 3D (multicellular spheroids) cell cultures, tissue explants and in human tumour xenograft experiments. Derivatives of the ureidobenzenesulfonamide and ureidosulfamate CAIX/XII chemical classes are inhibiting tumour cell proliferation, migration and invasion when used in the low micro molar concentration range. Studies in mice carrying either solid tumours or experimental metastases have shown that ureidobenzenesulfonamide and ureidosulfamate CAIX/XII inhibitors are well tolerated with no obvious negative effects on physical wellbeing and body weight. U-104 (SLC-0111), belonging to the ureidobenzenesulfonamide class of CAIX/XII inhibitor, successfully completed clinical Phase I and is currently in clinical Phase I/II for the treatment of metastatic pancreatic ductal cancer [13]. The chemical structures and Ki values for CAI, CAII, CAIX and CAXII of the sulfamate and benzenesulfonamide compounds mentioned in this review are shown in Figure 1.

## 2. Ureidosulfamate CAIX/XII Inhibitors in Preclinical Studies as a Single Therapy

Ureido-substituted sulfamates have gained significant attention over the last decade as small molecular weight inhibitors specifically targeting the tumour-associated CAIX and CAXII isoforms. A large group of ureidosulfamates CAIX/XII derivatives were studies as part of a large-scale collaborative EU-financed project called METOXIA [14]. As a single drug therapy, 4-(3′-(3″,5″-dimethylphenyl)ureido)phenyl sulfamate (S4) (10–125 mg/kg) did not reduce the growth of primary breast or laryngeal squamous cell tumours [15,16], yet significantly reduced metastatic progression in the orthotopic MDA-MB-231 xenograft model [15]. More recently, S4 drug therapy was validated on two different types of primary small cell lung cancer (SCLC) tumours [17]. The particular relevance of using SCLC cells in CAIX-based therapy became evident from a study with *CA9* gene knockdown in HT-1080 cells, showing differential regulation of multiple genes associated with SCLC signaling [5]. S4 (50 mg/kg) as a single agent reduced the growth of primary SCLC tumours derived from DMS 79 or COR-L24 cells. 4-(3′-(4″-Chlorophenyl)ureido)phenyl sulfamate (FC9-398A), an analogue of S4, with better stability in pharmacokinetic studies, showed a reduction in the primary growth of subcutaneous MDA-MB-231 xenografts [18]. It was noticed that S4 therapy influences the level of CAIX ectodomain (CAIX ECD) shedding [16]. The biological relevance of shedding CAIX ECD is still not well understood, despite the increase in attention from different research groups over recent years. It is thought that CAIX ECD from tumour cells in response to chemotherapy is likely to have a dual-action, in which the effectiveness of the cytotoxic drug is reflected by an apoptosis-associated increase in CAIX ECD shedding which can be used as an indicator for the chemotherapy response. On the other hand, CAIX ECD may contribute to paracrine signaling implicated in cancer progression [19]. Meyer and colleagues observed a reduction in CAIX ECD shedding into serum of laryngeal squamous cell carcinoma mice in response to S4 therapy [16]. The level of CAIX ECD shedding by colorectal carcinoma cells (CRC) was also influenced by S4 treatment, but depended on the type of CRC cell line used [20].

**Figure 2:**
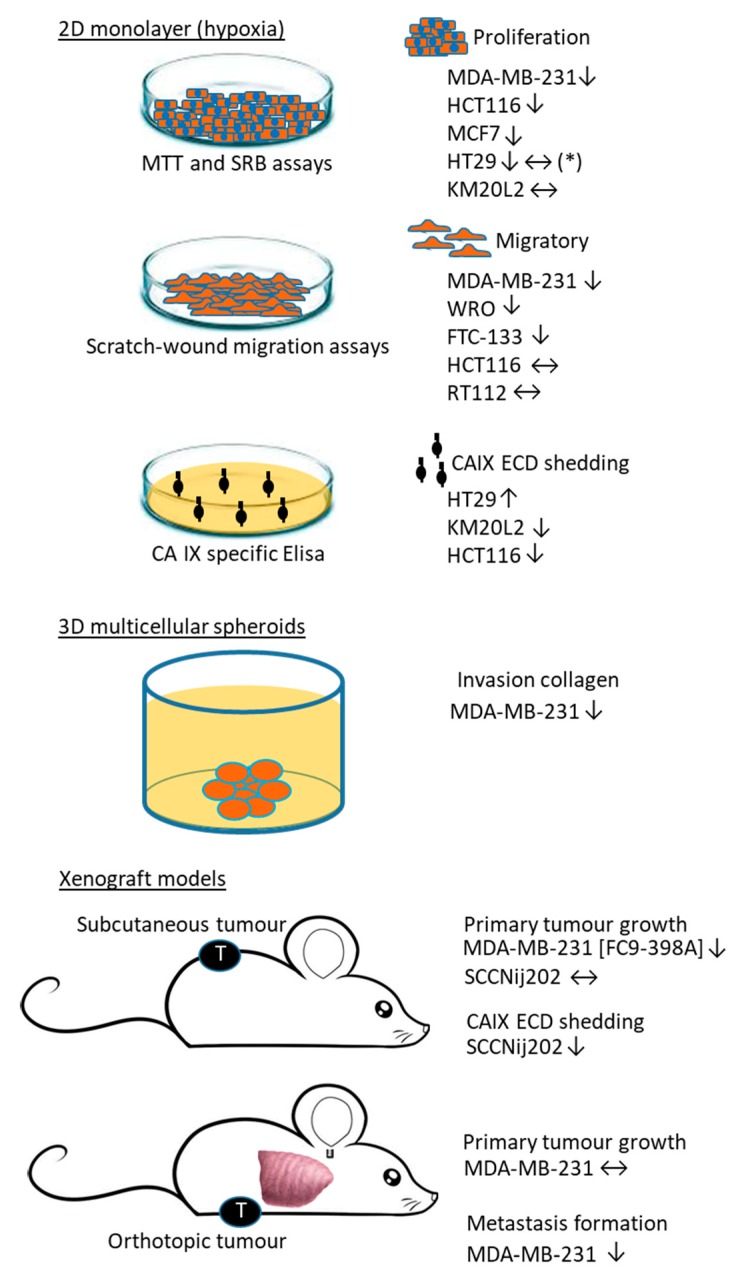
Summary of ureidosulfamate CAIX/XII studies to date. All data are on compound S4, or where indicated on FC9-398A. The effects on hypoxic cancer cells in cell culture studies and those in various xenograft models are either showing a reduction (↓), increase (↑) or no effects (↔). (*) Discrepancy between two studies [15,20].

pH regulatory enzymes have a central role in maintaining a slightly alkaline intracellular pH (pH_i_) in cancer cells, as these cells rely on glycolysis for their energy supply producing large amounts of protons and lactate [21]. There are various different types of plasma membrane-associated proteins involved in the regulation of the pH in solid tumours. The main proteins involved are the ATPases (in particular vacuolar type, V-ATPase) and Na^+^-H^+^ exchangers (particular type 1, NHE1) which are responsible for the extrusion of protons from cancer cells. In addition, the import of HCO_3_^−^ drives the release of protons through the Na^+^/HCO_3_^−^ co-transporter and Cl^−^/HCO_3_^−^ exchanger. CAIX and CAXII both enhance the acidification of the extracellular environment (pH_e_) through proton production and the facilitation of HCO_3_^−^ uptake and are pivotal in maintaining a slightly alkaline pHi through facilitation of CO_2_ diffusion and lactate release. Interfering with pH regulation in LS174Tr (adenocarcinoma) spheroids by silencing of *CA9* gene (LS-*shCA9*/*ctl*) reduced proliferation and the growth was further reduced when both *CA9* and *CA12* genes (LS-sh*CA9*/*CA12^−^*) were silenced. Xenograft tumours derived from LS-sh*CA9*/*ctl* or LS-sh*CA9*/*CA12^−^* cells showed a 40% and 85% reduced tumour growth, successively, as compared to controls [22]. These observations highlight the importance of targeting both CAIX and CAXII isoforms in targeted cancer therapies. Monocarboxylate transporters (MCTs) and in particular MCT1 and MCT4 facilitate H^+^-linked transport of lactate across the plasma membrane. Cooperation between these different proteins is pivotal to the pH regulation of hypoxic tumour cells. The proteoglycan-like (PG) domain of CAIX has a central role as facilitator of the proton-coupled lactate transport in hypoxic cancer cells, and targeting the PG domain reduced cell proliferation and migration [23]. Liskova and colleagues showed that CAIX binds to NHE1 and the sodium/calcium exchanger (NCX), regulating the pHi of hypoxic cancer cells. The NCX1/CAIX/NHE1 complex enhanced the acidification of the pHe and stimulated cellular migration [24]. The clinical importance of in particular CAIX, Na^+^-H^+^ NHE1 and V-ATPase, made them a valid target for anticancer therapeutic strategies. Meehan and colleagues showed that breast cancer cell lines MDA-MB-231, MCF-7 and HBL-100 are expressing CAIX, NHE1 and V-ATPase and treatment with S4 (CAIX inhibitor), DMA (NHE1 inhibitor) or bafilomycin A1 (V-ATPase inhibitor) reduced proliferation. Exposure to acute hypoxia (here 24–72 hours) resulted in a resistance to the antiproliferative response of these inhibitors [25]. The effect of S4 treatment on the level of CAIX expression was breast cancer cell line dependent, but changes in CAIX expression did not affect the expression of NHE1 and/or V-ATPase. S4 treatment was most effective in reducing the invasion of MDA-MB-231 and HBL-100 in collagen type I, suggesting that of these three pH regulatory proteins, CAIX is most important in facilitating invasion of breast cancer cells [25]. The acidification of the extracellular environment by the upregulation of CAIX and NHE1 in response to hypoxia leads to protonation of their inhibitors. The binding of sulfamate- and sulfonamide-type CAIX/XII inhibitors to the CAIX/XII isozymes is also likely affected by the acidity of the tumour environment, as protonation and deprotonation are an essential part of the linked reactions involved in the binding of these inhibitor to the CA active site. This may help to explain why the effect of S4 on the pHe of CAIX-positive CRC lines was more pronounced in CO_2_/HCO_3_^-^-free media as compared to CO_2_/HCO_3_^-^-buffered media [20]. The protonation of S4 and FC9-399A may also have accounted for the reduced potential of these inhibitors to induce cell death in Me30966 (melanoma) cells in CO_2_/HCO_3_^-^-free media as compared to CO_2_/HCO_3_^-^-buffered media [26]. Inhibitors targeting the pH regulatory enzymes CAIX, NHE1 and V-ATPase on breast cancer cells were also effective in reducing proliferation and invasion of CAIX-positive breast cancer cell lines in CO_2_/HCO_3_^-^-buffered media [25]. The main findings on the use of ureidosulfamate CAIX/XII inhibitors in preclinical studies are shown in Figure 2.

## 3. Ureidosulfamate CAIX/XII Inhibitors in Preclinical Studies in Combination with Cisplatin/Doxorubicin

Several research groups have also validated whether ureidosulfamate CAIX/XII inhibitors can be used to enhance the effectiveness of traditional chemotherapies, reduce common side effects and to postpone the development of chemotherapy resistance/treatment failure. These are all clinically relevant hurdles as chemotherapies are not usually a single treatment but a course of treatment, which includes a number of chemotherapy cycles. In this regard, whilst the use of cisplatin in patients with SCLC tumours is standard of care, acquired resistance is a well-recognised phenomenon. A common observation is that SCLC tumours respond well to cisplatin initially, but after several treatment cycles, the tumour cells become cisplatin-resistant, resulting in relapse [27]. Targeting hypoxia/CAIX as an approach to enhance the response of SCLC tumours to current treatment regimens was previously shown to be a viable novel strategy [28]. To mimic the clinical scenario of prolonged use of cisplatin, mice with primary DMS 79 and COR-L24 SCLC tumours received up to four cycles of either cisplatin alone or in combination with the sulfamate CAIX/XII inhibitor S4 [17]. Cisplatin/S4 combination therapy was more effective in inhibiting DMS 79 primary tumour growth, as compared to both agents alone. Importantly, the tumour response to cisplatin after the end of S4 treatment mirrored the response after a single treatment with cisplatin, indicating that repeated cisplatin doses did not result in a cisplatin-resistant cell population [17]. The cisplatin/S4 combination reduced the CAIX-positive cells in the DMS 79 tumours, suggesting a hypoxia-specific target.

The uptake of chemotherapeutic drugs very much depends on the pH outside of the tumour cells (pHe). In particular, the uptake by tumour cells of weak base drugs like doxorubicin is lowered due to the CAIX/XII-induced acidification of the extracellular environment. To combat the protonation of doxorubicin in the extracellular environment, we previously showed that inhibiting CA activity with acetazolamide, a non-isoform specific CA inhibitor, increased the uptake and cytotoxicity of doxorubicin in CAIX-overexpressing MDA-MB-435 (melanoma) cells [29]. The cytotoxic effect of combining doxorubicin with CAIX/XII inhibitor S4 in cells exposed to hypoxia is very much cell line dependent [30]. As there are, besides carbonic anhydrase, seven other major molecules involved in pH regulation of cancer cells (see discussion in previous paragraph), a significant level of adaptation to keep the intracellular pH (pHi) constant is likely to occur, in cancer cells targeted with CAIX/XII inhibitors. In line with this assumption, the combination of proton pump inhibitor (PPI) Lansoprazole, targeting the V-ATPase ion/proton pump, with a CAIX/XII inhibitor (S4 or FC9-399A), proved to be more effective than single treatments, in inhibiting proliferation and increasing apoptosis of Me30966 cells [26].

## 4. Ureidobenzenesulfonamide CAIX/XII Inhibitors in Preclinical and Clinical Studies

By means of X-ray crystallography and CA inhibition assays with hCAs I, II, IX and XII, a large number of ureidobenzenesulfonamide derivatives incorporating different R-ureido moieties have been tested for the selective binding to the enzyme (active site) of the tumour-associated over other CA isoforms [31]. Biological validation in specific in vitro assays for cytotoxicity, proliferation, migration and invasion, have identified several interesting compounds. Ureido-substituted benzenesulfonamide Compound 25 (4-([(3’-nitrophenyl)carbamoyl]amino) benzenesulfonamide) showed a strong anti-metastatic effect in the 4T1 (murine breast carcinoma) intravenous model [31]. Treating MDA-MB-231 and MCF-7 cells with U-104 led to a significant reduction in CAIX expression and activity, coinciding with a reduction in clonogenic survival, migration and cells in G0/G1 phase, and an increase in the level of apoptotic cells [32]. U-104 (SLC-0111) (4-[[[(4-fluorophenyl)amino]carbonyl]amino]-benzenesulfonamide) significantly inhibited primary tumour growth in the orthotopic MDA-MB-231 model and inhibited metastases formation in the 4T1 experimental metastasis model [33]. Treatment with U-104 also inhibited primary tumour growth in the orthotopic MDA-MB-231 and 4T1 breast carcinoma models, by targeting the cancer stem cell (CSC) population in a TORC1-dependent manner [34].

U-104 reduced the AT-1 (rat prostate carcinoma) cancer cell growth but did not enhance the cytotoxicity of chemotherapeutic drugs cisplatin or daunorubicin, a member of the same anthracycline antibiotics as doxorubicin [35]. U-104 sensitised cells to conventional chemotherapeutic agents such as dacarbazine and temozolomide (melanoma), doxorubicin (breast cancer) and 5-fluorouracil (colon cancer) [36]. In combination with chemotherapy drug paclitaxel, U-104 was able to target the Paclitaxel-resistant cells, reducing the primary tumour growth and metastasis formation. U-104 sensitised MDA-MB-231 and MCF-7 breast cancer cells to irradiation [32]. Carbohydrate-based sulfamates CAXII inhibitors (here compounds 1, 2 and 4) are highly effective in human cancer cell lines expressing different levels of CAXII and P-glycoprotein (Pgp), a drug efflux transporter which contributes to multidrug resistance. Inhibition of CAXII reversed the resistance of Pgp-expressing cancer cells for doxorubicin and reduced the cell viability [37]. Sulfamate FC9-403A sensitised MDA-MB-231 spheroids to irradiation, resulting in a significantly reduced number of colonies as compared with either drug treatment or irradiation alone [25]. Sulfamate S4 and irradiation had no combinatorial effect in 3D clonogenic assays [25]. U-104 (SLC-0111) completed a clinical Phase I (NCT02215850) and is currently in clinical Phase I/II trials in combination with gemcitabine in pancreatic cancer (NCT03450018). The clinical Phase I trial was designed to obtain data on safety, tolerability and pharmacokinetics of SLC-0111 in a single group of 24 patients with advanced or unresectable solid tumours. The results of the Phase I trial have yet to be published. The fact that SLC-0111 is being trialled in humans has induced the development of SLC-0111 analogues, tested for the potential to inhibit the tumour-associated CA isoforms (CAIX/XII) over the off-target CA isoforms (CA I/II), with the sulfanilamide ureido derivatives being highly effective and some being selective [38].

## 5. Carbonic Anhydrase IX/XII Inhibitor Studies and the Choice of Cancer Cell Line

A large variety of different murine and human cancer cell lines have been employed to validate the effectiveness of ureido-substituted benzenesulfonamide and sulfamate CAIX/XII inhibitors. These cell lines all derived from solid tumours that show upregulation of CAIX expression, associated with poor prognosis due to the high metastatic rate and/or the acquisition of treatment resistance. What most of these cancer cell lines have in common is no or a very low expression of CAIX in normoxic conditions, with significantly higher expression of CAIX under hypoxic conditions. There are several exceptions; HT29 colorectal carcinoma (CRC) cells have a high baseline expression of CAIX and both HCT116 (CRC) and RT112 (bladder carcinoma) are negative for CAIX in both normoxic and hypoxic conditions. Based on studies with native and *CA9* gene knockout in HT-1080cells, the differential expression of CAIX clearly affected SCLC signaling. On occasions where CAIX/XII inhibitors are used in combination with other therapies, there is often an additional reason for the use of a particular cell line (e.g., expression of V-ATPase on Me30966). The human MDA-MB-231 and murine 4T1 triple negative breast cancer cell lines have been favoured for several reasons. Both cell lines are highly metastatic, which allows study of the effectiveness of CAIX/XII inhibitors on localised cancer growth as well as on metastases, the latter only when cancer cells are injected in the mammary fat pad (orthotopic location) or intravenously injected (experimental metastasis). The use of MDA-MB-231 cells tagged with enhanced green fluorescence protein (eGFP-MDA-MB-231 cells) or 4T1 cells expressing luciferase (bioluminescence) allowed for the identification and quantification of cancer cell migration and colony formation in vitro and metastasis formation in vivo [15,31]. One note of caution in the use of MDA-MB-231 and MCF-7 cells in carbonic anhydrase inhibitor (CAI) studies. In addition to the expression of CAIX activity on the plasma membrane (the default expression pattern), a robust CAIX activity is present in the cytosol of MDA-MB-231 and MCF-7 cells [39]. In our research, confocal microscopy analysis of CAIX expression (M75 antibody) in MDA-MB-231 and HT-1080 cells revealed also a partial cytosolic localisation of CAIX in MDA-MB-231 cells versus a complete localisation on the plasma membrane for the HT-1080 cells (data not shown). It is tempting to speculate that at least part of the differences in sensitivity between cell lines for the CAIX/XII selective inhibitors is due to the cellular localisation of the corresponding isozymes.

## 6. Biological Validation of Ureido-Type Sulfamate and Benzenesulfonamide CAIX/XII Inhibitors

The mammalian alpha family of carbonic anhydrases (α-CAs) consists of 16 members, with all except CAXV expressed in human tissues. Clinically used drugs that target α-CAs like acetazolamide, methazolamide and dichlorophenamide are primarily used to treat glaucoma [40], and can also be used to treat seizure disorder, acute mountain sickness and gout. The drawback of most of these clinically available CAI is that they are not CA isoform-specific, and often target CA isoforms expressed in healthy tissues. In the last decade or so, this recognition has triggered significant effort towards the synthesis and biological validation of novel isoform-specific CAI, including those selective for the tumour-associated CAIX/XII isoforms. Stopped-flow CO_2_ hydration assays are used to validate the potential of CAI to inhibit the different human CA isoforms. Ureido-type sulfamate and benzenesulfonamide CAIX/XII inhibitors are screened for the potential to inhibit the tumour-associated hCAIX/XII isoforms over the off-target hCAI/II isoforms. The most effective inhibitors have an inhibitory constant (Ki) for inhibition of hCAIX/XII in the low nanomolar range. In recent years, many novel sulfamate and sulfonamide CAIX/XII inhibitors have been generated by synthetic chemists, and in particular by the research group of Professor Supuran. Changes to the ureido-linker, the “spacer” between the benzene sulfamate / benzenesulfonamide Zinc Binding Group (ZBG) and the highly variable tail region included incorporating piperazinyl-ureido moieties in a large series of sulfamate CA inhibitors [41] and sulfonamide CA inhibitors [42]. Analogues based on SLC-0111, the single CAIX/XII inhibitor currently in clinical trial, are selective CAIX/XII inhibitors and inhibited proliferation of HT29, MDA-MB-231 and PC-3 human cancer cell lines [43]. The validation of novel CA inhibitors in cell-free systems, like the stopped-flow CO_2_ hydration assays, is normally succeeded by biological validation in cell-based systems of the most effective CAIX/XII inhibitors. There are several important aspects to the biological validation of sulfamate and sulfonamide CAIX/XII inhibitors in cell-based systems. There are a range of cell viability assays used which measure different things. The 3-(4,5-dimethylthiazol-2-yl)-2,5-diphenyltetrazolium bromide (MTT) and Sulforhodamine B (SRB) assays measure effects on proliferation, whereas the CellTiter-Glo assay measures adenosine triphosphate (ATP) levels in the cell. Cell death (apoptosis) is measured in different ways (e.g., TUNEL) which do not always discriminate between the effects of CAIX/XII inhibitors on early and late stages of apoptosis. It was, for instance, noticed that sulfonamide CAIX inhibitor A1 predominantly increased the population of early-stage apoptotic HeLa (cervix carcinoma) cells or late-stage apoptotic HeLa cells depending on the concentration of inhibitor in the culture media [44].

## 7. Target Cell Populations of Ureido-Type Sulfamate and Benzenesulfonamide Inhibitors

Several research papers have dealt with the question of which subpopulations of cancer cells are targeted by sulfamate and benzenesulfonamide CAIX/XII inhibitors. In particular, therapies to target the treatment-resistant populations associated with hypoxic niches are much sought after. Some CAIX/XII-selective compounds based on the sulfamate or benzenesulfonamide scaffold inhibit proliferation and increase apoptosis of different cancer cells in normoxic conditions [18,45] or more prominent under hypoxic conditions [15,17,32,46]. The increased apoptosis is associated with a reduced expression of CAIX, suggesting that apoptosis is induced in high CAIX expressing cells. In mouse tumour models, the reduced growth in tumour cells targeted by selective CAIX/XII-based therapies coincides with areas with high levels of apoptosis and necrosis [17,18]. Ferroptosis, a form of regulated necrosis, is part of the response to blocking CAIX activity with S4 [46]. These hypoxic niches are also home to cancer stem cells (CSCs) and mature cancer cells undergoing epithelial-to-mesenchymal transition (EMT) which are both associated with resistance to therapy and cancer progression. Benezenesulfonamide CAIX/XII inhibitor U-104 diminished the CSC population (EpCAM+ cells) in primary MDA-MB-231 tumours [34]. In addition, U-104 in combination with temozolomide reduced the number of brain tumour-initiating cells, revealed by the reduction in CD133+ expression (stem cell marker) and the capacity of neurosphere formation [47].

CAIX/XII-specific inhibitors target metastatic disease in the MDA-MB-231 and 4T1 breast cancer models [15,31,33]. Circulating tumour cells (CTCs) from MDA-MB-231 tumours respond differently to hypoxia than parental cells in the expression of specific hypoxia/HIF1-regulated genes like *CA9* [48]. A lower hypoxia-induced expression of CAIX in CTCs may help to evade recognition by CAIX/XII-selective inhibitors. CAIX in the murine mammary (4T1) adenocarcinoma is involved in the development of metastasis by the production of granulocyte colony secreted factor (G-CSF), which is stimulating the recruitment of bone marrow-derived cells (BMDC), including the immunomodulatory myeloid-derived suppressor cells (MDSCs) to the pre-metastatic niches [49].

## 8. Conclusions

Much of the progress on the use of CAIX/XII-selective inhibitors, both from a therapeutic and biological mechanism point of view, comes from the class of ureidosulfonamides and in particular from U-104/SLC-0111. However, as this review shows, preclinical work on the ureidosulfamate class of CAIX/XII-selective inhibitors is encouraging and worthy of further investigation. In particular, a better understanding of the relationship between cancer cell type and variable sensitivity for these inhibitors is needed to identify the tumours most likely to benefit in clinical studies. Furthermore, which common chemotherapeutic agents are most likely to benefit from combination therapy with the ureidosulfamate class CAIX/XII inhibitors, and whether we are truly able to target the chemotherapy-resistant subpopulation requires elucidation. The low bioavailability of compounds like S4 also needs further investigation. One of the contributing factors, in the case of S4, is the insufficient solubility in aqueous media. Formulation strategies (e.g., lipid-based carrier systems) or synthesis technology (e.g., water soluble analogues and prodrugs) may improve the bioavailability. There may be a significant drug candidate within the already available CAIX/XII-selective inhibitors. Cooperative working with pharmaceutical and biotech companies may enable appropriate development of programmes towards clinical candidate validation from the current lead preclinical ureidosulfamate CAIX/XII inhibitors.

## Abbreviations

CAI	Carbonic anhydrase inhibitor
CAs	Carbonic anhydrases
CSCs	Cancer stem cells
CTCs	Circulating tumour cells
ECD	Ectodomain
HIF-1	Hypoxia inducible factor 1
HREs	Hypoxia-response elements
PPI	Proton pump inhibitor
SCLC	Small cell lung cancer
TNBC	Triple-negative breast cancer
